# Rethinking COVID-19 Vulnerability: A Call for LGBTQ+ Im/migrant Health Equity in the United States During and After a Pandemic

**DOI:** 10.1089/heq.2020.0012

**Published:** 2020-05-27

**Authors:** Nolan S. Kline

**Affiliations:** Department of Anthropology, Rollins College, Winter Park, Florida, USA.

**Keywords:** LGBTQ+ health, immigrant health, COVID-19, intersectionality

## Abstract

Public health responses to the coronavirus disease 2019 (COVID-19) pandemic have emphasized older adults' vulnerability, but this obfuscates the social and political root causes of health inequity. To advance health equity during a novel communicable disease outbreak, public health practitioners must continue to be attentive to social and political circumstances that inform poor health. Such efforts are especially needed for populations who are exposed to numerous social and political factors that structure health inequity, such as lesbian, gay, bisexual, transgender, or otherwise-queer identifying (LGBTQ+) populations and im/migrant populations. The COVID-19 outbreak is, therefore, a critical time to emphasize root causes of health inequity.

## Introduction

In December of 2019, health officials in China reported a cluster of patients with a novel respiratory illness that became known as coronavirus disease 2019 (COVID-19).^1^ By March 11, 2020, the disease had spread to 114 countries and the World Health Organization declared COVID-19 a pandemic.^2^ In the United States, the COVID-19 response comprised a patchwork approach of local and state governments attempting to mitigate outbreaks and reduce community transmission. At the time of this writing, the ongoing COVID-19 outbreak resulted in the United States having the largest number of confirmed COVID-19 cases globally,^3^ and 23 states had issued orders urging the public to stay home.^4^ Moreover, the U.S. economy, like the global economy, saw dramatic volatility and record-breaking unemployment claims, sparking concerns about a COVID-19–related recession.^5^ In response to the pandemic, public health messages about which populations would be most impacted by COVID-19 largely focused on adults aged 65 years and older due to high mortality rates.^6^ However, exclusive emphasis on older adults as an at-risk population ignores other populations who are vulnerable to disease—particularly those who encounter a number of social and political factors that structure existing health inequity, such as lesbian, gay, bisexual, transgender, or otherwise-queer identifying (LGBTQ+) populations and im/migrant populations. In addition to encountering several social factors that structure inequity, these populations have historically been stigmatized during outbreaks of novel communicable diseases such as AIDS, severe acute respiratory syndrome (SARS), and H1N1.^7–9^ Accordingly, to advance health equity during a pandemic and a time in flux when a novel communicable disease has distorted social norms, public health practitioners must continue sustained attention to social and political circumstances that inform poor health for LGBTQ+ populations, im/migrants, and individuals with intersecting LGBTQ+ im/migrant identities.

## COVID-19 and Health-Related Vulnerability

U.S. public health officials have stressed that older adults are a particularly high-risk group for COVID-19–related morbidity and mortality. The Centers for Disease Control and Prevention (CDC) specifically underscored that people over the age of 65 years were “at higher risk for severe illness,” and that 8 out of 10 COVID-19–related deaths in the United States were among adults aged 65 years and older.^10^ Although attention to older adults' risk is important, emphasizing older adults' vulnerability nevertheless obfuscates social and political factors that undergird existing health inequalities for other populations. These existing social and political factors can become exacerbated during communicable disease pandemics, such as the COVID-19 outbreak unfolding at the time of writing this article.

## LGBTQ+ Health Inequalities

LGBTQ+-identifying people experience a number of health-related vulnerabilities rooted in social and political inequalities.^9,11^ LGBTQ+ people face barriers to culturally competent health care and may encounter social stigma regarding their identity, sexual orientation, or gender expression in clinical spaces.^12^ Compared with cisgender heterosexuals, LGBTQ+ populations are more likely to lack health insurance and rate their health as poor.^9,13^ Furthermore, LGBTQ+ populations have historically carried social stigma associated with AIDS since the first cases of AIDS were reported in white gay men.^9^ Although LGBTQ+ populations may not be stigmatized by COVID-19, the pandemic may nevertheless disproportionately impact LGBTQ+ individuals due to existing social and political inequalities.

Political inequalities play a substantial role in LGBTQ+ populations' COVID-19–related vulnerabilities. For example, as of the time of this writing, LGBTQ+ people in the United States lack federal employment protections from being fired because of their sexual orientation. This policy vacuum makes LGBTQ+ populations especially susceptible to employment loss during the pandemic and concomitant economic downturn resulting in widespread unemployment. Income loss structures housing insecurity, and some LGBTQ+ populations experience heightened housing instability compared with cisgender heterosexual peers.^14^ The pandemic may, therefore, exacerbate existing employment and housing inequalities. Furthermore, the COVID-19 pandemic could disproportionately affect LGBTQ+ individuals' mental health since some LGBTQ+ groups have elevated risk of depression and substance abuse,^15,16^ and recommended social distancing measures associated with COVID-19 may aggravate depressive symptoms and complicate access to support groups and treatment services.

## Im/migrant Health Inequalities

Like sexual orientation and gender identity, immigration status is a source of social vulnerability. Immigration status is a social determinant of health^17^ that can determine eligibility for public benefits and structure access to health care. Im/migrants with precarious immigration statuses, including those who are undocumented, typically lack employer-provided health insurance^18^ and may engage in labor with significant occupational health risks. Undocumented laborers may also work in essential services, such as construction and agricultural industries, and, therefore, may have constrained ability to follow social distancing guidelines. As a result, they may face higher likelihood of exposure to COVID-19 because their work does not allow for sustained social distancing.

In addition, U.S. immigration policies can have deleterious consequences for some immigrants, including undocumented Latinx populations.^19,20^ This is noteworthy during the COVID-19 pandemic because the Trump administration has championed aggressive immigration policies, including expanding “public charge” definitions and ending reprieves from deportation by terminating the deferred action for childhood arrivals program.^21,22^ Although Immigration and Customs Enforcement (ICE) officials announced they would halt raids and enforcement actions near health facilitites,^23^ enforcement actions continue elsewhere, and the Trump administration announced immigration restrictions in response to the pandemic, including a temporary suspension on issuing permanent resident cards.

Communicable disease outbreaks can heighten social factors that structure health inequalities, such as racism and xenophobia. For example, the 2003 SARS epidemic resulted in widespread blaming of “Chinese culture” for the disease and greater acceptance of health disparities among immigrants.^7^ Similarly, the H1N1 influenza outbreak in 2009 and the 2015–2016 Zika virus epidemic contributed to blaming and otherizing Latin Americans.^8,24^ The COVID-19 pandemic demonstrates that history repeats itself. As of writing this article, President Trump and other federal government officials have referred to COVID-19 as the “Chinese Virus,” using rhetoric that otherizes immigrants and perpetuates blame.

## Intersectional Inequalities: LGBTQ+ Im/migrants During a Pandemic

People with intersecting identities, such as being a sexual and gender minority and a racial and ethnic minority, experience multiple overlapping forms of social marginalization that may be heightened during a pandemic. This is particularly true for LGBTQ+ im/migrants who also have precarious immigration statuses and identify as “undocuqueer.” Their unique social locations make them susceptible to interlocking forms of oppression such as xenophobia, racism, and discrimination based on their sex, sexual orientation, gender expression, or gender identity.

These intersecting forms of marginalization structure health-related vulnerabilities that can be worsened during an infectious disease pandemic. For example, because LGBTQ+ populations lack federal employment protections based on their sexual and gender identities, they may be at elevated risk for unemployment during the pandemic, particularly those who work in the service economy. For LGBTQ+ populations who are undocumented or have undocumented family members, this situation is compounded by the fact that households with undocumented immigrants are excluded from financial relief offered through the Coronavirus Aid, Relief, and Economic Security Act. This unique social location, therefore, creates a form of economic vulnerability that structures poor health.

Economic insecurities for people with intersecting LGBTQ+, racial and ethnic identities, and immigration statuses can deepen health-related vulnerability. Subsets of these populations relying on specific medications, such as pre-exposure prophylaxis, may encounter additional financial constraints to secure their medications and may also experience heightened levels of food insecurity and housing instability. Because of their complex relationship with large-scale social factors, these vulnerabilities are not always visible, and the COVID-19 pandemic may perpetuate hidden health inequalities that demand continued action.

## Action for Health Equity During a Pandemic

The COVID-19 pandemic has provided insight for ways to advance health equity during a global health crisis. First, public health discussions of vulnerability in the United States must extend beyond populations with greatest threats of mortality and must be attentive to the social and political factors that structure poor health. In addition, some actions taken during a pandemic—such as ICE not targeting hospitals or clinics—should be a norm, not an exception during an outbreak, and ending aggressive immigration tactics should persist beyond the COVID-19 crisis as a way to advance health equity. The COVID-19 pandemic further reveals how specific legal protections—such as employment nondiscrimination policies—are important public health policies demanding scholarly and political attention. In addition, the COVID-19 pandemic provides insight regarding the importance of social solidarity and activism during infectious disease outbreaks. The HIV/AIDS activist group, ACT UP, demonstrated such solidarity in response to President Trump referring to COVID-19 as “the Chinese Virus” and asserting a need to “close borders,” when they tweeted: “We remember a time when HIV/AIDS was called ‘gay-related immune deficiency’ aka GRID and ‘gay cancer’. NEVER AGAIN will we let world leaders transfer blame to communities. Trust science over discrimination always.”^25^ ACT UP's message emphasizes an ongoing need for solidarity to advance health equity for all populations.

## Conclusion

Focusing on LGBTQ+ and im/migrant populations' vulnerabilities during the COVID-19 pandemic is not meant to ignore vulnerability among other populations. This emphasis underscores the importance of continued attention to social and political factors that structure poor health and demonstrates the need for attention to these issues during and after a pandemic. Accordingly, the COVID-19 outbreak represents a critical time to emphasize root causes of health inequity.

## Abbreviations Used

CDC: Centers for Disease Control and Prevention
COVID-19: coronavirus disease 2019
ICE: Immigration and Customs Enforcement
LGBTQ+: lesbian, gay, bisexual, transgender, or otherwise-queer identifying
SARS: severe acute respiratory syndrome
